# *In Vivo* Pyro-SIP Assessing Active Gut Microbiota of the Cotton Leafworm, *Spodoptera littoralis*

**DOI:** 10.1371/journal.pone.0085948

**Published:** 2014-01-27

**Authors:** Yongqi Shao, Erika Arias-Cordero, Huijuan Guo, Stefan Bartram, Wilhelm Boland

**Affiliations:** Department of Bioorganic Chemistry, Max Planck Institute for Chemical Ecology, Jena, Germany; University of Liverpool, United Kingdom

## Abstract

The gut microbiota is of crucial importance for the host with considerable metabolic activity. Although great efforts have been made toward characterizing microbial diversity, measuring components' metabolic activity surprisingly hasn't kept pace. Here we combined pyrosequencing of amplified 16S rRNA genes with *in vivo* stable isotope probing (Pyro-SIP) to unmask metabolically active bacteria in the gut of cotton leafworm (*Spodoptera littoralis*), a polyphagous insect herbivore that consumes large amounts of plant material in a short time, liberating abundant glucose in the alimentary canal as a most important carbon and energy source for both host and active gut bacteria. With ^13^C glucose as the trophic link, Pyro-SIP revealed that a relatively simple but distinctive gut microbiota co-developed with the host, both metabolic activity and composition shifting throughout larval stages. *Pantoea*, *Citrobacter* and *Clostridium* were particularly active in early-instar, likely the core functional populations linked to nutritional upgrading. *Enterococcus* was the single predominant genus in the community, and it was essentially stable and metabolically active in the larval lifespan. Based on that *Enterococci* formed biofilm-like layers on the gut epithelium and that the isolated strains showed antimicrobial properties, *Enterococcus* may be able to establish a colonization resistance effect in the gut against potentially harmful microbes from outside. Not only does this establish the first in-depth inventory of the gut microbiota of a model organism from the mostly phytophagous Lepidoptera, but this pilot study shows that Pyro-SIP can rapidly gain insight into the gut microbiota's metabolic activity with high resolution and high precision.

## Introduction

The gut microbiota, largely composed of bacteria, is a complex ecosystem, and forms close symbiotic associations with the host. Not only thriving in the gut, these inner microbial residents also contribute to host metabolism through nutrient release, xenobiotic detoxification and immune regulation, all of which greatly boost host fitness [1], [2]. With the rapid development of new generation sequencing technologies, a growing number of studies from humans to ants, have demonstrated the fascinating microbial diversity and composition within the gut [3], [4]. However, the assessment of metabolically active components in the gut microbiota to date is surprisingly scarce, particularly under the host physiology. Clearly, not all microorganisms are able to colonize the gut, even though it is a nutrient-rich habitat, as some dietary microbes are lysed and some transients remain dormant during gut passage [2]. On the other hand, active populations constantly shift in response to host development and environmental effects, and hence metabolic potentials delineated by pure metagenomic analysis have to be verified *in vivo* [5]. To get a more complete picture, it is important to move beyond a mainly sequencing-based approach towards other advanced tools to identify active fractions of the community that directly contribute to the current function of the microbiota.

Stable isotope probing (SIP) is a promising, culture-free technique that is often used in environmental microbiology to identify active microorganisms involved in various biogeochemical processes [6], [7]. This methodology relies on the assimilation of a stable isotope (^13^C)-labeled carbon source into growing microbes and the selective recovery of the isotope-enriched cellular components, such as the most informative nucleic acids, which could provide specific taxonomic information. ^13^C-enriched “heavy” DNA or RNA can be separated from unlabeled, normal “light” (^12^C) nucleic acids by density-gradient ultracentrifugation and subsequently retrieved from gradient fractionation procedure for further molecular analysis [8]. Stable isotope probing of nucleic acids provides direct evidence of bacterial metabolic activity and has been demonstrated to be more sensitive than an RNA-based approach [9]. Recently Pilloni *et al.* introduced a combination of DNA-SIP and pyrosequencing, namely Pyro-SIP, in SIP gradient interpretation [10]. However, this study only performed pyrosequencing on the entire metagenomic DNA without gradient separation and still relied on the low-resolution gel electrophoresis-based fingerprinting (T-RFLP) and laborious clone library construction to identify active bacteria. Considering all of those drawbacks, here we directly pursue pyrosequencing to examine separated gradient fractions to get more resolution, which is relatively straightforward and effectively merges the microbiota structure investigation with a measurement of local metabolic activity.

As a first attempt, we successfully conducted this refined Pyro-SIP to unravel the metabolically active bacterial community in the gut of an insect model organism, *Spodoptera littoralis* (Lepidoptera, Noctuidae), a highly polyphagous pest found worldwide that causes considerable yield losses of many economically important crops. The devastating larval stage of *S. littoralis* (cotton leafworm) consumes large amounts of plant material in a short time, liberating abundant plant-derived saccharides mainly glucose in the alimentary canal to be a most important carbon and energy source for both host and active gut bacteria. More recently, the microbiology of this important insect has received increasing attention, a complex and variable commensal microbiota is found in the larval gut [11] and gut bacteria are suggested to be involved in multitrophic interactions between plant, herbivore and predator, which is previously underestimated [12]–[14].

Using ^13^C glucose as the trophic link, Pyro-SIP revealed that a relatively simple but distinctive gut microbiota co-develops with the host, both metabolic activity and composition shifting throughout larval stages. Three families, including *Enterococcaceae*, *Clostridiaceae* and *Enterobacteriaceae*, are particularly rich and active inside, which likely represent the core functional populations in the gut linked to nutritional upgrading and pathogen defense for improving larval fitness. This study also establishes the first in-depth inventory of the gut microbiota of a model organism from mostly phytophagous Lepidoptera. Knowledge of the gut bacteria of such a major insect herbivore could lead to new targets for pest control.

## Results

### Identification and Quantification of Saccharides in the Gut Content

Sugar composition was analyzed in different gut regions, namely the foregut, midgut and hindgut. Gut contents collected from larvae fed on their native diet (cotton) or defined artificial diet (without antibiotics or preservatives) were analyzed by gas chromatography-mass spectrometry (GC-MS) after water extraction and aldononitrile acetate derivatization, which is a rapid and sensitive derivatization method for non-volatile saccharides and each sugar forms a single chromatographic peak after GC separation [15], [16].

**Figure 1:**
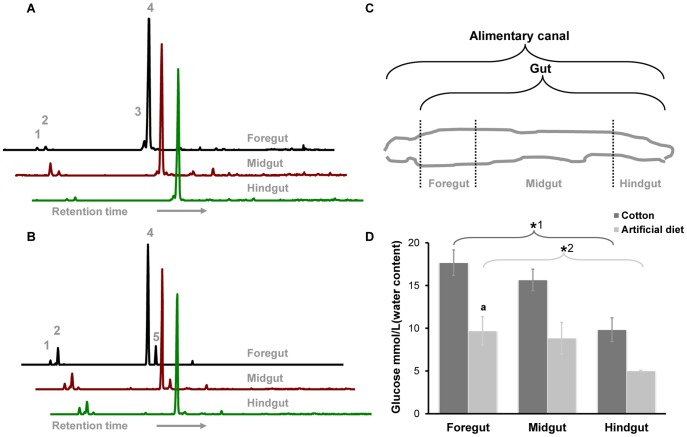
Sugar composition in the gut content of cotton leafworm. (**A**) Sugars in the gut content of larvae fed on cotton or (**B**) artificial diet by GC-MS characterization after aldononitrile acetate derivatization. The larval alimentary canal is divided into three regions: foregut, midgut and hindgut, shown in diagram (**C**). (**D**) Quantification of dominant glucose reveals a significant decrease in average content along the gut. However, cotton-feeding larvae exhibit higher amount of glucose in all gut regions. * and **^a^** indicate significant difference: P (* ^1^) = 0.0020, P (* ^2^) = 0.0366, P (^a^) = 0.0017. Error bars indicate standard errors. 1, Ribose; 2, Arabinose; 3, Mannose; 4, Glucose; 5, Galactose.

The GC-MS profile indicated that several pentose and hexose sugars were present in the gut, including ribose, arabinose, mannose, glucose and galactose (Fig. 1A and B). However, glucose was dominant in all portions of the gut and other monomers at much lower concentrations. The similar pattern of sugar monomer composition in a given larval section was detected in every replicate. After acid hydrolysis, the number and concentration of detectable monosaccharides increased, with glucose and arabinose being dominant, reflecting various soluble polysaccharides in the gut (Figs. S1 and S2).

As the major sugar, glucose was quantified based on a specific enzymatic assay, which revealed that maximal glucose concentrations occurred in the foregut and then dropped from the anterior to posterior end of the gut. In cotton-feeding larvae, the average concentration of glucose in the aqueous phase of the foregut was approximately 17.7 mM, significantly higher than that in the hindgut, which was around 9.8 mM (Fig. 1D). The rapid decrease of glucose from the foregut to the hindgut along the short alimentary canal indicated that glucose was remarkably consumed during gut passage. The same trend was obtained with larvae fed on artificial diet. However, the mean values of glucose concentration in all gut regions of larvae fed on artificial diet were lower than those of larvae fed on cotton (Fig. 1D).

### *In Vivo* Labeling Strategy with ^13^C Glucose as the Trophic Link

As the most effective energy and carbon source available, nutritive glucose in the gut provided a good chance to carry out *in vivo* SIP by performing ^13^C-glucose amendment. Initial concentrations of glucose in the foregut of larvae fed on cotton reached 20 mM, but only about 10 mM in larvae fed on artificial diet (Fig. 1D). Thus, the artificial diet was amended with 10 mM exogenous glucose to mimic the *in situ* concentration of glucose in larvae fed on cotton. In order to track metabolically active bacteria, fully ^13^C-labeled glucose was supplemented as the labeling treatment, whereas the same amount of native-glucose (^12^C) was used in the control group. We tested the labeling process by feeding early-instar larvae (from 1^st^ to 2^nd^) on glucose-amended artificial diets for 24 and 48 hours, respectively. For revealing the difference during larval development, the same process was also employed in the study of late-instar larvae (5^th^).

**Figure 2:**
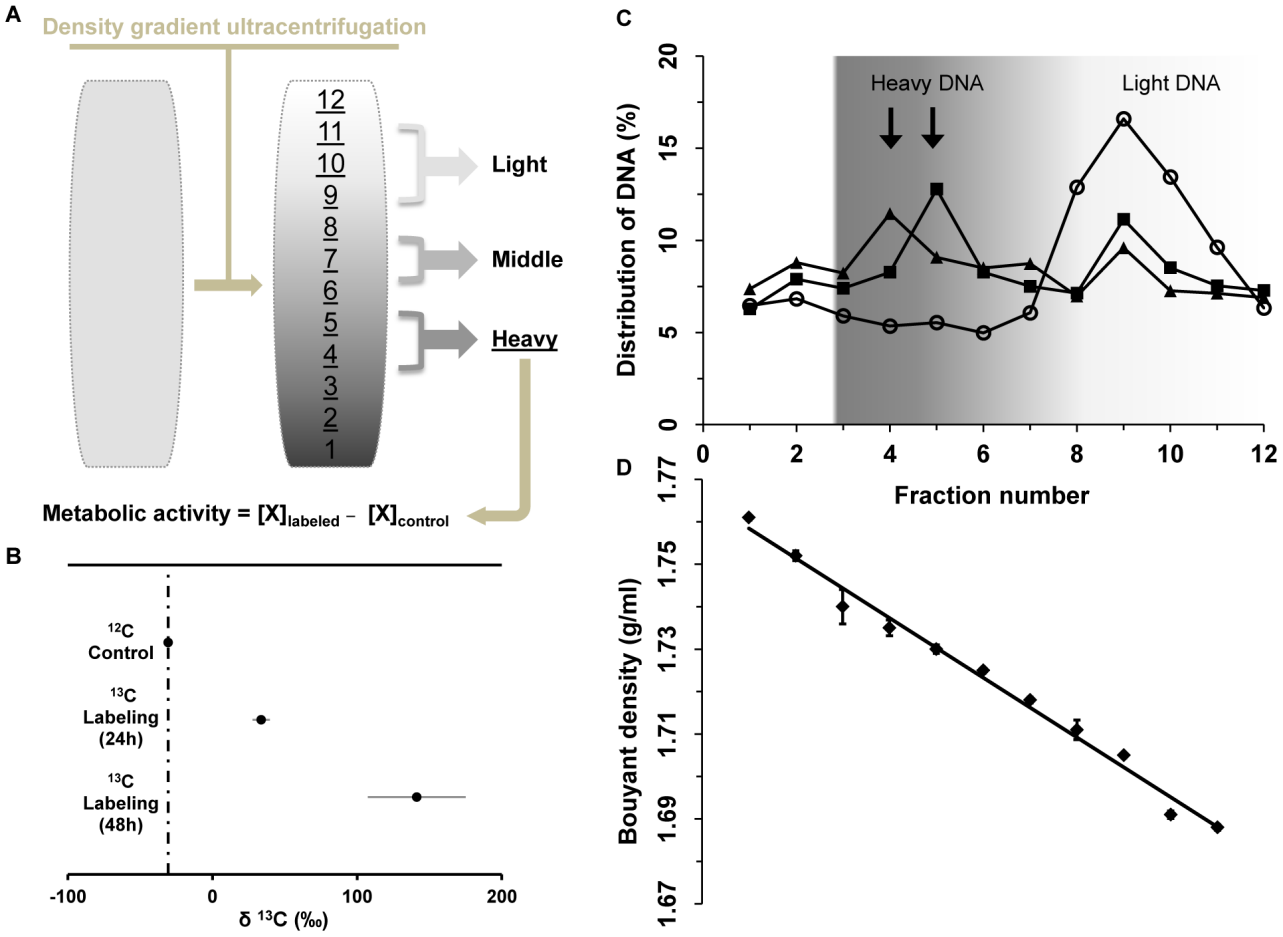
Profiling of active bacteria. (**A**) For determining active bacteria, ^13^C-labeled DNA is separated by density-gradient ultracentrifugation and subsequently retrieved from representative fractions, with darker color indicating labeled heavy DNA. All separated DNA samples (light, middle and heavy) were directly subjected to quantitative pyrosequencing for revealing species lineage and relative abundance. For each taxon **X**, metabolic activity is calculated as the difference in relative abundance between heavy fraction of the labeled sample and that of the control. (**B**) Isotopic ratio (δ^13^C) of DNA samples. (**C**) Distribution of DNA content in gradient fractions of glucose treatments. Symbols: **O**, DNA extracted from the [^12^C]-glucose control; ▪, DNA extracted from the [^13^C]-glucose treatment for 24 h; **▴**, DNA extracted from the [^13^C]-glucose treatment for 48 h. Arrows indicate that considerable ^13^C-labeled DNA shifted to heavy gradients, compared with the control. (**D**) Gradient fraction analysis by density after 40 h centrifugation of a 1.725 g ml^−1^ starting CsCl solution. Error bars indicate standard errors.

Larvae taken from both the ^13^C treatment and unlabeled control group were dissected at each time point, and total DNA was extracted from fresh gut tissues. Isotopic ratio mass spectrometry (IRMS) measurements of δ^13^C in the extracted DNA showed substantial isotope enrichment in both 24 h (δ^13^C = 33.6±5.3‰) and 48 h (δ^13^C = 141.2±33.1‰) labeling samples, compared to natural stable isotope abundance in the control (δ^13^C = −30.7±1.3‰) (Fig. 2B). Feeding for 48 h enhanced the labeling process with the isotopic shift exceeding 100‰ (P = 0.0008), indicating that abundant newly-divided bacterial cells had already replaced old ones. Considering that a large amount of insect DNA was co-extracted from the gut tissue, the real proportion of ^13^C in pure bacterial DNA will be even higher, which is suitable for density gradient centrifugation to separate labeled nucleic acids and identify active community members.

### Density Gradient Ultracentrifugation and Recovery of Separated DNA

Both the labeled and control samples were conducted in the same batch of isopycnic ultracentrifugation to minimize potential variation during this process. After 40 h ultracentrifugation, formed gradients were fractionated into 12 equal aliquots, and the abundance of DNA present in each individual fraction was quantified, allowing us to compare DNA template distribution in the formed gradients (Fig. 2A). The density of all fractions from the control and labeled samples was checked by weighing, covering an average gradient from 1.688 g ml^−1^ to 1.761 g ml^−1^, which was in the expected range according to previous reports [8]. Density determination revealed a linear trend from the bottom to the top, indicating proper gradient formation (Fig. 2D).

After 24 h feeding, labeled DNA from metabolically active bacteria was measurable in the ^13^C treatment, which had a new peak of abundant DNA at the buoyant density (BD) of 1.730 g ml^−1^ (fraction 5). After 48 h, the peak shifted to an even heavier fraction at BD of 1.735 g ml^−1^ (fraction 4), indicating more ^13^C incorporation into DNA, which confirmed the IRMS data. By contrast, most of DNA from the unlabeled control was still distributed over light fractions (BD was <1.718 g ml^−1^) and no peak shifted towards the high BD (Fig. 2C). Notably, a low background of unspecific nucleic acids was detected in all gradient fractions, which is common in the environmental SIP experiment [17]. Since the 48 h-feeding sample generated a significantly higher level of enrichment and a larger shift in BD ensured an efficient separation of isotopically labeled DNA from the unlabeled DNA, samples from this treatment were selected for the downstream molecular characterization of microbial community.

16S rRNA gene was amplified with a general bacterial primer from each gradient fraction. The amplification of control gradients yielded apparent PCR products from fractions 8 to 12, which is in line with the DNA smear formed after ultracentrifugation, and some weak bands in the heavy fractions, which were expected from unspecific background DNA. In contrast, the labeled sample displayed increased band intensity in the related heavy fractions (3–6), which was caused by the increased amount of ^13^C-DNA template. Next, 16S rDNA amplicons detected in gradient fractions were analyzed by denaturing gradient gel electrophoresis (DGGE) in order to fingerprint the community. Changes became visible when the DGGE profile derived from the ^13^C treatment was compared with that from the unlabeled control. Several strong bands were clearly detected from heavy fractions (3–6) of the labeled sample, whereas almost no pattern changed over all gradients of the control (Fig. S3). Those unique patterns associated with heavy fractions from the stable isotope-amended sample but not from the native substrate-amended control provided strong evidence linking certain organisms with the *in situ* gut metabolic activity. Taken together, the data corroborated the successful incorporation of ^13^C into the DNA of active gut bacteria.

Based on the fingerprinting analysis, we combined fractions with BDs ranging from 1.730 to 1.735 g ml^−1^ into a compiled “heavy” fraction. BDs ranged around 1.718 g ml^−1^ in a “middle” fraction and from 1.688 to 1.705 g ml^−1^ in a “light” fraction for subsequent pyrosequencing.

### General Microbial Structure in the Gutflora and Phylogenetic Analysis

Quantitative pyrosequencing was performed directly on representative SIP gradients to reveal species lineage and relative abundance. PCR amplification using archaea- or fungus-specific primers failed to amplify any archaeal 16S rRNA or fungal ITS sequences. Cultivation-based attempts to recover fungi from the gut with three general fungus-growing agar plates were also unsuccessful (Fig. S4).

**Figure 3:**
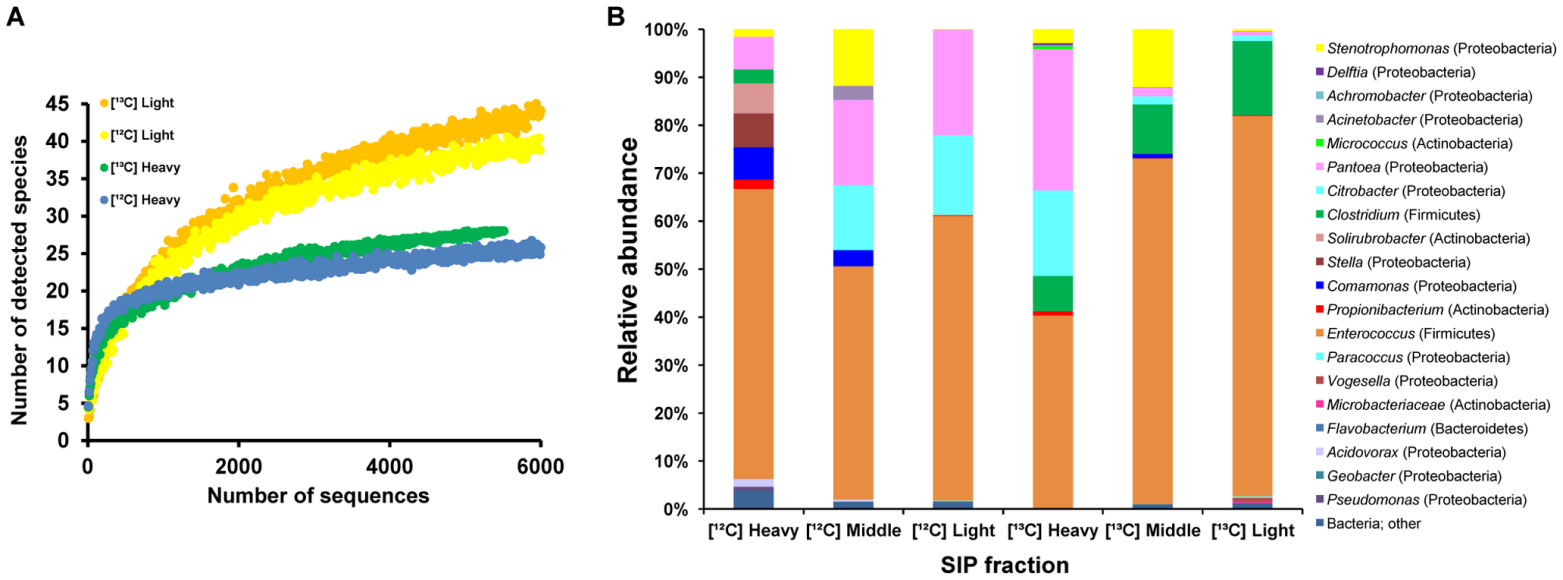
Bacterial diversity and relative abundance in the gut microbiota of early-instar larvae. (**A**) Rarefaction curves of 16S rDNA sequences were obtained from representative SIP fractions of the control ([^12^C]) and labeling treatment ([^13^C]). (**B**) Relative abundance of bacterial taxa in different SIP fractions, represented in a relative area graph as revealed by pyrosequencing. Abbreviations: [^12^C] Light, light fractions (fractions 9–11, Fig. 2A) of native-glucose amendment; [^12^C] Middle, middle fraction (fraction 7) of that; [^12^C] Heavy, heavy fractions (fractions 4–5) of that; [^13^C] Light, light fractions of ^13^C-glucose amendment; [^13^C] Middle, middle fraction of that; [^13^C] Heavy, heavy fractions of that.

**Figure 4:**
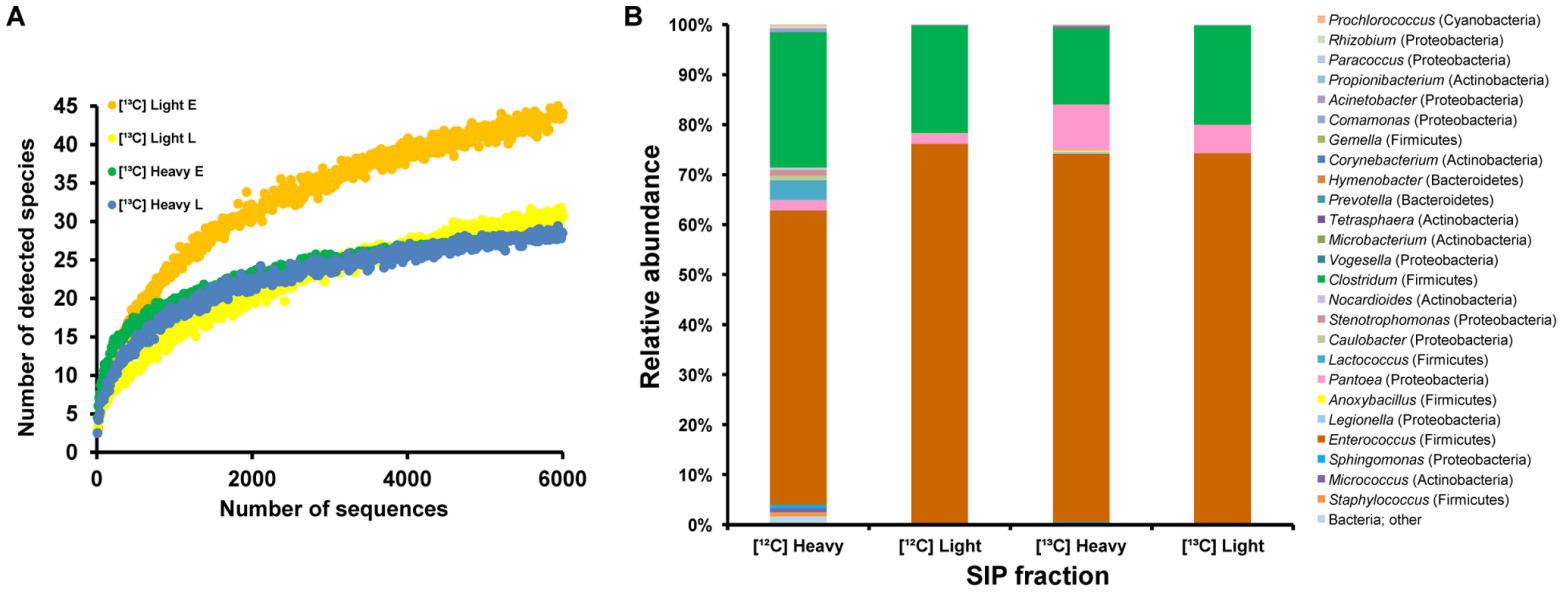
Bacterial diversity and relative abundance in the gut microbiota of late-instar larvae. (**A**) Rarefaction curves of 16S rDNA sequences were obtained from representative SIP fractions of early-instar and late-instar larvae. Abbreviations: E, representative fractions from early-instar larvae fed on ^13^C-glucose; L, fractions from late-instar larvae fed on ^13^C-glucose. (**B**) Relative abundance of bacterial taxa in different SIP fractions, represented in a relative area graph as revealed by pyrosequencing. Abbreviations are the same as in Fig. 3.

Bacteria-specific primer for pyrosequencing amplified the V1–V3 region of 16S rRNA gene for taxonomic classification. After denoising and removing chimeric sequences, 120,045 high quality reads were generated, with an average length of 404 nucleotides. There was a low incidence of sequences unclassified at the phylum level (maximum 3.82%). For all samples, the rarefaction curve tended towards saturation at similar numbers of sequences at the species level, indicating the sampling was comprehensive (Figs. 3A and 4A). Various methods were employed to accurately estimate the diversity of individual samples (Table 1). The gut microbiota of cotton leafworm was taxonomically restricted, which contained 22–42 operational taxonomic units (OTUs) detected at 97% sequence similarity, with Proteobacteria and Firmicutes being co-dominant phyla. The Shannon index of diversity at 1.46–2.48 was the low boundary of the diversity in soils (2.4–3.6) [18].

**Table 1:** Richness and diversity estimate of the 16S rRNA gene from the pyrosequencing analysis.

Sample	Species richness indices		Species diversity indices	
	Observed	PD tree	Shannon	Simpson
**Early-instar control**				
Light fraction	34	3	1.85	0.60
Middle fraction	26	2	2.48	0.72
Heavy fraction	23	3	2.43	0.64
**Early-instar labeled**				
Light fraction	38	3	1.24	0.36
Middle fraction	22	2	0.16	0.47
Heavy fraction	27	2	2.57	0.75
**Late-instar control**				
Light fraction	23	3	1.46	0.50
Heavy fraction	42	4	2.36	0.67
**Late-instar labeled**				
Light fraction	24	2	1.57	0.52
Heavy fraction	25	3	1.66	0.51

The “light” fraction collected from unlabeled controls, where most metagenomic DNA still distributed in, served for profiling the majority of the bacterial community. Due to fewer DNA templates from dominant groups being translocated to the heavy fraction of the control, some less-abundant bacteria, such as *Comamonas* and *Stella*, had more chance to be amplified and showed an increased proportion in the profile of [^12^C]-Heavy fraction (Fig. 3B). Therefore, the comprehensive analysis of all fractions from the unlabeled SIP control provided detailed information about the overall community diversity, which is largely similar with that of larvae fed on the white bean diet, without DNA ultracentrifugation (Fig. S6). The clearly decreased abundance of dominant groups, like *Pantoea* and *Citrobacter*, in the middle and heavy fraction, caused by fewer target sequences, furthermore reflected some quantitative properties of pyrosequencing [19]. In total, eleven phyla were identified by the RDP classifier from the dataset, but only five displaying a relative abundance larger than 0.1% in at least one of the analyzed samples (Table 2). Firmicutes apparently dominated both larval developmental stages, which represented 59.2% of the number of sequences of early-instar larvae and 97.2% of late-instar.

**Table 2:** Abundance of the 16S rRNA gene in each larval stage at phylum level.

% of total sequence reads in each developmental stage		
Phylum	Early-instar larvae	Late-instar larvae
**Actinobacteria**	1.28	0.11
**Acidobacteria**	0.18	0.00
**Bacteroidetes**	0.00	0.11
**Firmicutes**	59.23	97.21
**Proteobacteria**	38.88	2.21
**Other**	0.43	0.36

**Figure 5:**
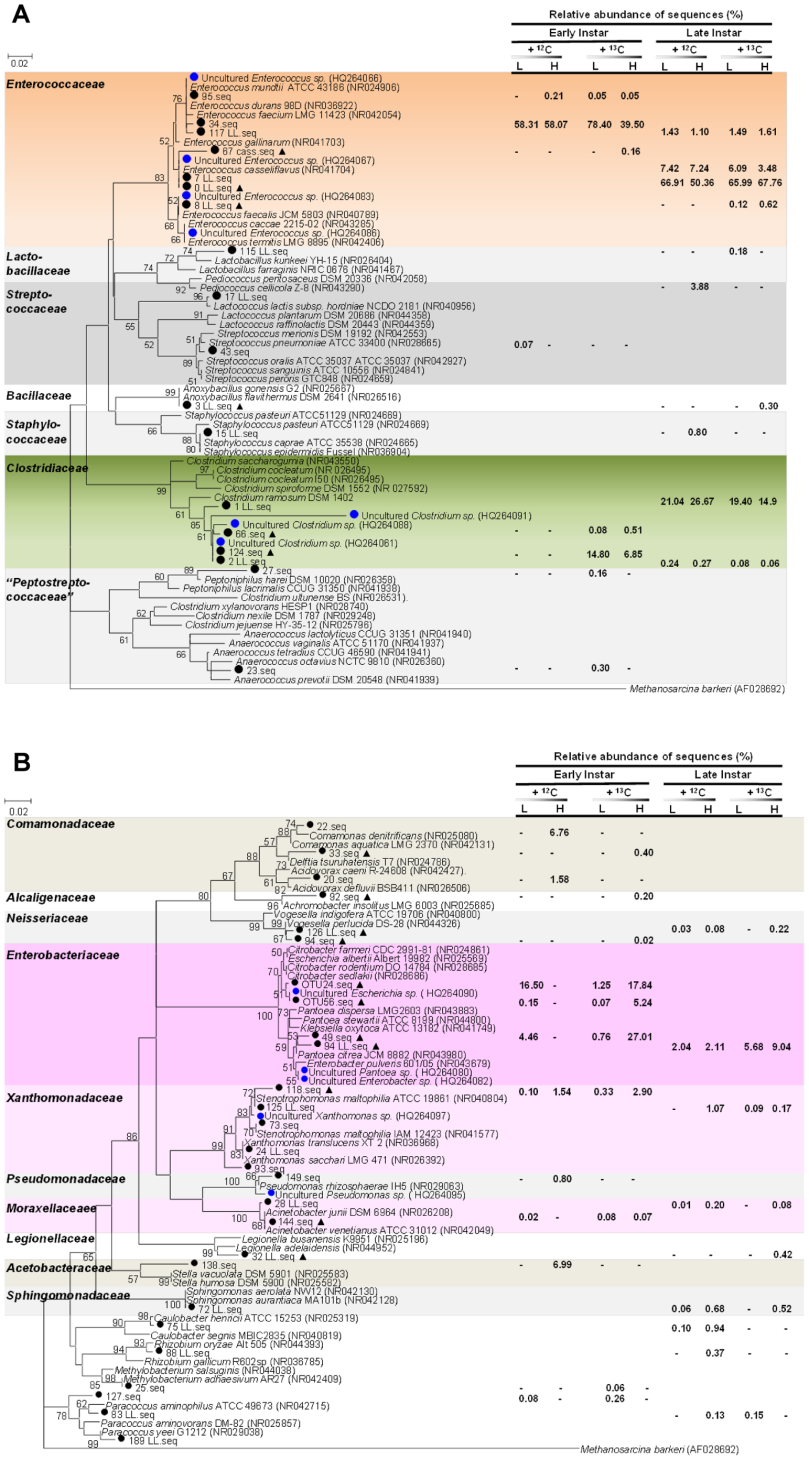
Phylogenetic analysis of (a) Firmicutes and (b) Proteobacteria identified from the gut microbiota of cotton leafworm. (**A**) Maximum Likelihood tree was derived from partial 16S rDNA sequence data for members of Firmicutes. (**B**) Neighbor-Joining tree was derived from partial 16S rDNA sequence data for members of Proteobacteria. Representative pyrosequences from this work and near full-length 16S rDNA sequences retrieved from previous clone-library-based studies are indicated by black circles (•) and blue circles, respectively. Labeled taxa are marked with triangles (▴). Reference sequences are downloaded from GenBank (accession numbers are in parentheses.). *Methanosarcina barkeri* (AF028692) is used as an outgroup. Family-level clusters are indicated by different colors. Bootstrap values (in percent) are based on 1000 replications. Bar represents 2% sequence divergence. Right section denotes percentage of representative bacterial 16S rRNA sequences in the total dataset of each sample. Abbreviations: +^12^C, native-glucose amendment; +^13^C, ^13^C-glucose amendment; L, light fractions; H, heavy fractions.

Proteobacteria was another major phylum in early-instar larvae, accounting for 38.9% of total sequences. Bacteria in this phylum are especially wide-spread in herbivore microbiotas and often affiliated with insect symbionts. Most dominant OTUs corresponded to *Pantoea citrea* and *Citrobacter farmeri*, which represented 17.2% and 16.4% of the reads, respectively, and belonged to the *Enterobacteriaceae* family of the Gammaproteobacteria class ([^12^C] light, Fig. 3B). In addition to *Enterobacteriaceae*, other Gammaproteobacteria from the family *Xanthomonadaceae*, *Pseudomonadaceae* and *Moraxellaceaee* were detected, including populations related to *Stenotrophomonas*, *Pseudomonas* and *Acinetobacter* (Figs. 3b and 5b). Other classes of Proteobacteria, such as *Paracoccus* from Alphaproteobacteria and *Geobacter* from Deltaproteobacteria, were also identified. Organisms from the phylum Actinobacteria, including the genera *Micrococcus*, *Solirubrobacter* and *Propionibacterium*, and a novel group, GP2, from the phylum Acidobacteria, made up a small fraction of reads. However, those rare phylotypes contributed to the richness of the community.

Firmicutes were represented by the family *Enterococcaceae*, comprising >50% of all sequences. The major OTU was closely associated with *Enterococcus mundtii* as determined by Mega BLAST with the representative sequence. Other *Enterococcus* species with a low identification score, such as *E. casseliflavus*, were found too, but these accounted only for a small fraction. A low number of *Clostridia* were also retrieved from early-instar larvae.

Gut microbiota underwent a drastic change in late-instar, principally characterized by decreased species richness and diversity (Table 1). The rarefaction curve was significantly lower than that obtained in early-instar larvae (Fig. 4A). Firmicutes absolutely dominated the entire community, comprising >97% of all reads; simultaneously, sequences from other phyla decreased ([^12^C]-light, Fig. 4B). Pyrosequencing reads belonging to the *Clostridiaceae* family strongly increased: 21% of total sequences were affiliated with *Clostridium* species. *Enterococcus* still predominated, making up 75% of reads. Further bacteria of Firmicutes, such as members from the genera *Lactobacillus*, *Lactococcus*, *Anoxybacillus* and *Staphylococcus*, were detected too (Fig. 5A).

However, in the context of Proteobacteria, late-instar larvae mainly reduced the proportion of Gammaproteobacteria. *Pantoea* was detected at a small proportion with only 1.8% of total sequences ([^12^C]-light, Fig. 4B). *Citrobacter* quickly decreased together. In addition to common *Paracoccus*, more Alphaproteobacteria, such as members from the genera *Sphingomonas*, *Caulobacter* and *Rhizobium*, were detected. But at the same time *Stella* and *Methylobacterium* were removed. Betaproteobacteria disappeared from late-instar larvae, except for *Vogesella*. Other phylogenetic groups affiliating with the genera *Prevotella* (Bacteroidetes), *Micrococcus* and *Propionibacterium* (Actinobacteria) appeared in a small number of sequences.

Phylogenetic analysis showed that some pyrosequencing reads clustered together with the near full length of 16S sequences retrieved from other clone-library-based studies, suggesting common taxa present in *S. littoralis* (Fig. 5). Notably, the gut microbiota composition in larva fed on this artificial diet spiked with physiological dose of sugar was similar with that fed on its native plant diet [11]. Many low-abundant taxa were uncovered from our large-scale pyrosequencing, which more accurately represented the overall microbial community and also supplied sufficient taxonomic resolution.

### Characterization of Metabolically Active Bacteria in the Community

The “heavy” fractions collected from the labeled sample containing significant quantities of ^13^C-enriched DNA, and those collected from the unlabeled control served for profiling active populations in the community. Based on previous research, the metabolic activity of bacteria was assessed by calculating the difference between the relative abundance of individual taxa in the heavy fraction of the labeled sample and that of the control (Fig. 2A) [20], [21]. Moreover, involving a proper control in the SIP analysis subtracted the impact of the background ^12^C-DNA contamination in the heavy fraction, which ensured that bacterial diversity and abundance appearing or disappearing was not artifacts of the method itself. The rarefaction curve of the “heavy” fraction sample was below that of the “light” fraction because the active bacteria group was a subset of the total community (Fig. 3A).

**Figure 6:**
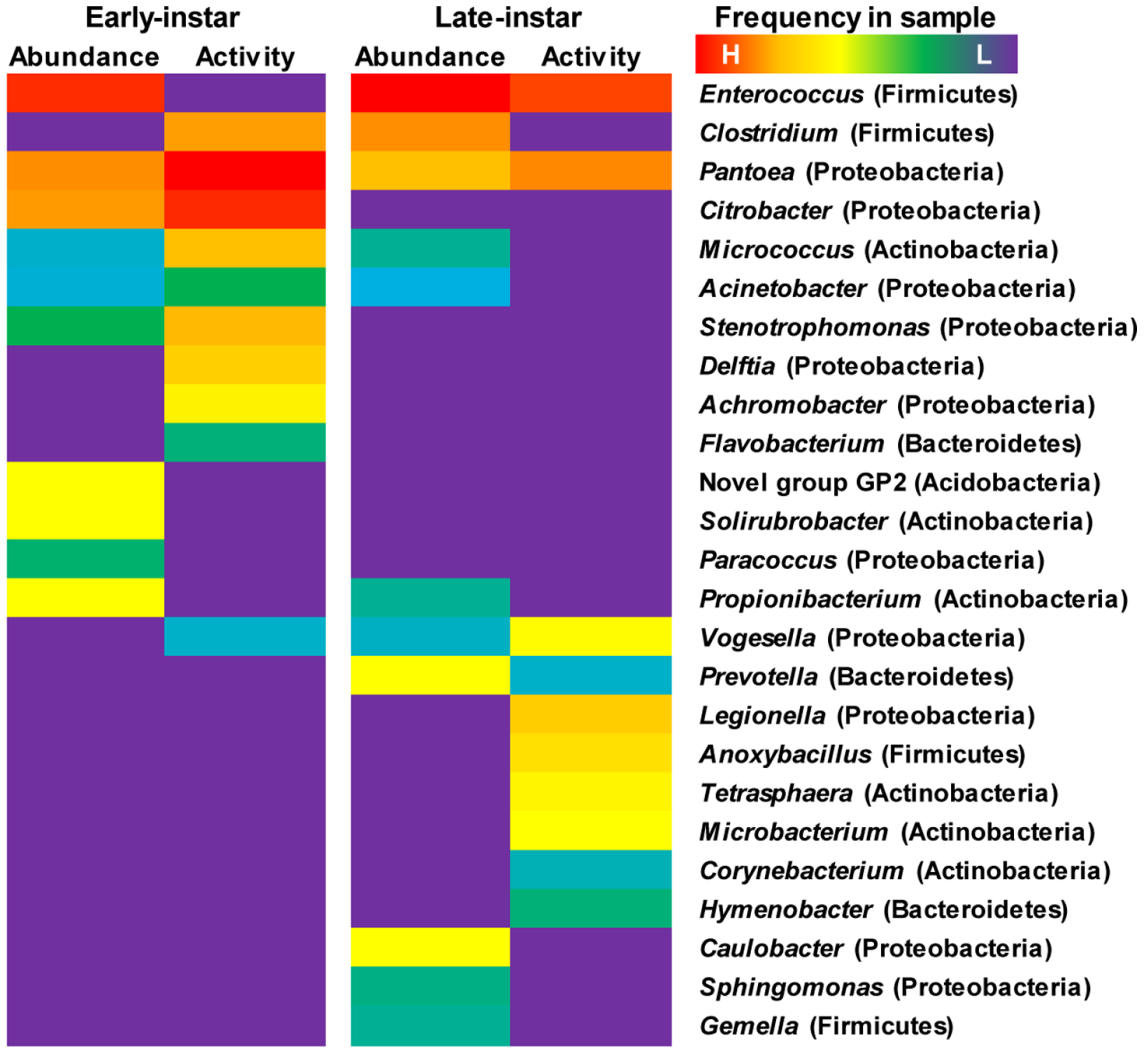
Frequency of 16S rRNA sequences in the microbiota obtained from the native-glucose control (bacterial relative abundance) and [^13^C]-glucose treatment (bacterial metabolic activity), represented as a heatmap. Left panel displays dynamic changes of taxa in early-instar larvae and right panel for late-instar. Warm colors indicate higher and cold colors lower abundance, calculated according to the formula in Fig. 2A (also see Fig. 5 for the percentage of taxa).

In early-instar, the pyrosequencing profile of the heavy fraction from the labeled sample ([^13^C]-Heavy, Fig. 3B) showed a largely increased abundance of certain species including *Pantoea*, *Citrobacter* and *Clostridium* by the METASTATS analysis (P<0.05), while negligible portions of these sequences were detected from the heavy fraction of the control ([^12^C]-Heavy, Fig. 3B). Therefore DNA from these bacteria was significantly labeled and they were considered to be metabolically active. The highest activity was assessed for *Pantoea* and *Citrobacter*, with a stimulation factor above 10% (Fig. 6). A diverse array of other Proteobacteria from the genera *Acinetobacter*, *Stenotrophomonas*, *Delftia*, *Achromobacter* and *Vogesella* also showed some activity according to their slightly increased intensity in the heavy fraction. *Clostridium* was another group which showed high metabolic activity although it was less abundant at this stage. The closely related phylotypes of these active species have been commonly identified from herbivore guts and are well-known plant biomass degraders. *Enterococcus* was abundantly present in all SIP fractions independent of the condition, but the number of sequences peaked in the middle fraction of the labeled sample compared with that of the control, indicating that it was metabolically less active, DNA from which was less incorporated with ^13^C and could not completely migrate to the heavy fraction. Members from genera such as *Paracoccus*, *Solirubrobacter* and *Propionibacterium* were neither enriched nor detected in the ^13^C-DNA fraction, and thus were considered metabolically inactive bacteria.

In late-instar, *Enterococcus* was more abundant in the heavy fraction of the labeled sample than in that of the control, being the most active member within the community. Fluorescence *in situ* hybridization (FISH) revealed a large amount of *Enterococci* closely adhered to the mucosal layer of gut epithelium, where they formed a thick biofilm-like structure (Fig. S5a and b). Interestingly, *Enterococcus* isolates can produce novel antimicrobial compounds against other bacteria (Fig. S5c and d). *Clostridium* sp. stayed at a high population level, which was the consequence of its high activity in early-instar; however, there was little or no metabolic activity at this stage. *Pantoea* and another Proteobacteria, *Vogesella*, were actively persistent in both developmental stages. In contrast, *Citrobacter* was not detected. But the genus *Legionella* was particularly active. Bacteroidetes, including *Prevotella* and *Hymenobacter*, showed some metabolic activity. Other populations were detected to be active, including *Tetrasphaera*, *Microbacterium* and *Corynebacterium* associated with the phylum Actinobacteria, and *Anoxybacillus* of Firmicutes.

Collectively, *in situ* SIP denoted different patterns of metabolic activity found in the gut microbiota during larval development. A consortium of *Enterobacteriaceae* (especially *Pantoea*, *Citrobacter*) and *Clostridium* apparently were more active in early-instar larvae, while *Enterococcus* became strongly active in fully grown late-instar larvae (Fig. 6). Those changes may directly associate with their functional roles inside the gut.

## Discussion

Although SIP has been frequently used in environmental microbiology, very few researches have applied this valuable technique to the study of gut microbiota. Those pioneering researches have almost exclusively pursued *in vitro* experiment systems to mimic the gut environment, which obviously could not fully duplicate true physiological conditions in the intact gut, especially host factors shaping the microbial community, and hence may be limited or biased [5], [20].

In contrast, we directly studied the gut microbiota of cotton leafworm *in vivo* by coupling pyrosequencing and SIP approaches. Cotton leafworms possess a simple tube-like alimentary tract, which is the largest part of the whole body and lacks any specialized substructures (Fig. 1C). Despite its simplicity, a large amount of bacteria, exceeding 10^7^ mL^−1^ [22], has been found occupying the gut and a high concentration of nutritive glucose is liberated there, making the gut an ideal environment for diverse microbial activities, including fermentation. These properties make this organism an ideal naturally-occurring model in which to study digestive-tract microbial symbiosis. As an effective and ubiquitous energy and carbon source, glucose provides a useful trophic link to identify metabolically active gut bacteria by tracing their assimilation of amended ^13^C-glucose in the artificial diet. The gut contains other non-labeled carbon sources, such as amino acids, which could be utilized by microbes. However those metabolically active bacteria should simultaneously consume the dominant common glucose too and thus are being labeled. The near *in situ* glucose concentration and relatively short labeling time ensured the original gut community would not be influenced by the SIP approach, as explained in detail elsewhere [20]. Cross-feeding is usually a major constraint in interpreting SIP experiments, which is not a problem in the present study since we aimed to capture the entire active community and active bacteria most likely are involved in the trophic network *in situ* [23]. DNA obtained from ^13^C-glucose treatments became enriched with ^13^C on the basis of a discernible DNA shift toward higher BDs; in contrast, no shift was observed with native-glucose amended controls. The substantial labeling of DNA was also validated by complementary methods, including isotopic ratio mass spectrometry measurement and fingerprint analysis. *In vivo* SIP provides key information about which groups are currently involved in gut metabolism, and these active bacterial associates potentially contribute to host fitness. The large sampling depth of pyrosequencing warranted the effective diversity survey, and rarefaction analysis indicated that our dataset afforded a sufficient degree of coverage for all samples.

Comprehensive analysis of the recovered DNA revealed a relatively simple but distinctive gut microbiota that co-develops with the host; both the composition and metabolic activity sustainably shift throughout larval stages. Overall, pyrosequencing reads were dominated by taxa from Proteobacteria and Firmicutes. A similar pattern has recently been described in fruitfly larvae [24]. Notably bacteria from these phyla are widespread in the gut of turtle ants, bees, moths and butterflies, suggesting that they may represent the chiefly phyla in insect herbivore gut microbiota [25]–[27]. The simplicity of this community, comprised of 22–42 OTUs, is especially apparent compared to the gut microbiota of insects from orders such as termites of Isoptera, or vertebrates, which often harbor hundreds of phylotypes [28], [29]. Strong alkalinity in the gut, considered an important determinant of community structure in beetle larvae, could also be the case in *Spodoptera*, which has a midgut pH >10 [22], [30]. Other key factors, including a fast food throughput and immune system function, may also account for this taxonomically restricted gutflora [31]. However, three families, including *Enterococcaceae*, *Clostridiaceae* and *Enterobacteriaceae*, are particularly rich and active, and these are likely the core functional populations living inside the gut. Furthermore a clear developmental change (early-instar vs. late-instar) is found. Not only do they consistently occur throughout the whole larval lifespan, these bacteria are also persistently identified from different sampling batches of normal larvae, indicating they are the indigenous gut residents and may be the true symbionts of *S. littoralis*.

In early-instar, a diverse assembly of Proteobacteria, especially Gammaproteobacteria dominated by *Pantoea* and *Citrobacter* from the family *Enterobacteriaceae*, was the most active group. Bacteria in this phylum closely associate with insect herbivores and possess broad polysaccharide-degrading abilities [32]–[34]. For example, cultivation-based studies reported *Enterobacteriaceae* isolated from silkworm (*Bombyx mori*) gut had the ability to secrete enzymes important in the digestion of complex dietary plant biopolymers such as cellulose, xylan, and pectin [32]. Genomic analysis of a *Pantoea* strain from a woodwasp (*Sirex noctilio*) detected genes putatively encoding for carbohydrate-active enzymes, with the majority predicted to be active on hemicellulose and more simple sugars [33]. Those *in vitro* growth assays and genomic investigation provide an initial picture of potential metabolic capabilities but do not offer information on bacterial activity *in situ*. Our Pyro-SIP results are the direct evidence of microbial metabolic activity *in vivo*, supporting the previous hypothesis that Gammaproteobacterial symbionts are involved in carbohydrate degradation. In spite of its low titer, *Clostridium* was another highly active group in early-instar larvae; these obligate anaerobes are capable of forming endospores. The colonization of *Clostridia* has commonly been linked to its highly efficient cellulose digestion and its ability to ferment a variety of sugars [35]. It can be assumed that the bacteria associated with cotton leafworm play a similar role. In addition, *Clostridia* may enhance host immunity [36]. Taking together, we suggest that the most active *Enterobacteriaceae* and *Clostridiaceae* synergistically participate in the digestive process in the gut, facilitating the breakdown of organic substrates in the foliage and making them more suitable for the host's digestion, absorption and metabolism. Given that sufficient nutrient supply is important in the early life, the released simple sugars would directly benefit early larval development, which further could be fermented to various other nutrients such as short-chain fat acids, greatly promoting the gut ecological environment. These bacteria, widely distributed in nature, could be easily acquired when larvae forage. The large sampling depth of this study revealed a number of rare taxa in the gut. Although this variety diminished over time, it is possible that rare members perform some of the microbiota's functions or have a role in special situations, as suggested by previous studies [37]. Further work need to be done to decipher their potential biological functions in the gut.

In late-instar, the gut microbiota became even simpler with a large decrease of microbial diversity and richness. More than 97% of community members belonged to Firmicutes, partially because of a significant *Clostridium* proliferation. *Enterococcus* predominated and was detected as the most active group. The successful expansion of Firmicutes over time probably in turn suppressed the growth of bacteria from other phyla in the same habitat, particularly Proteobacteria. Linked with larval development, changes in insect physiology such as dropped redox potential and enhanced host immunological response could be potential driving factors for observed changes. As larvae grow bigger, less oxygen can penetrate into the gut lumen over the thicker gut wall and elongated alimentary tract, which cause consistently low oxygen tension in the gut compartment [11]. This largely anoxic condition probably promotes the development of obligate anaerobic *Clostridium* and facultative anaerobic *Enterococcus*.

*Enterococcus*, a Gram-positive lactic acid bacterium (LAB), is the single predominant genus in the community. LAB are well-recognized beneficial organisms of the gut microbiota of many animals, including insects [38]. *Enterococci* are essentially stable throughout *Spodoptera*'s lifespan and are vertically transmitted from mother to offspring via egg, as shown for *Manduca* [9]. Therefore, they are the first to gain access to the gut and can immediately dominate there after hatching. As the founder species, *Enterococcus* likely controls the whole microbiota together with the host, which may be crucial for establishing a distinctive and reoccurring gut community. Besides in cotton leafworm, *Enterococcus* is present in diverse Lepidoptera, such as the larva of gypsy moth (*Lymantria dispar*) and cotton bollworm (*Helicoverpa armigera*) from both field and laboratory-reared samples [39], [40]. Notably, *Enterococcus* was found to be already active on the eggs of tobacco hornworm (*Manduca sexta*) based on rRNA detection [9]. These intimate associations indicate that *Enterococcus* may have important functional implications for Lepidoptera in general. FISH indicated that a high number of *Enterococci* closely attach to the mucus layer of gut epithelium to form a biofilm-like structure, which may be the reason for its high stability in the gut of healthy larvae and, furthermore, may prevent the gut from being invaded by harmful pathogenic microbes [34], [41]. Besides providing a physical barrier, isolated strains showed strong inhibition against other bacteria. A novel antimicrobial peptide was identified from the broth culture (Shao, unpublished data). Collectively, these findings indicate that *Enterococcus* is likely a defensive symbiont for the health of the host. Considering cotton leafworm is a generalist feeder in the field, its digestive tract is constantly challenged by the potentially harmful bacteria and fungal endophytes that it ingests. *Enterococci*, maintained in biofilm-like structure and showing potent antimicrobial properties, may be able to establish a colonization resistance effect in the gut, which protects the host against pathogens and a wide range of noncommensal competing microbes from outside [2], [42]. However, the well established *Enterococci* do not need fully turn on their metabolic machinery in a semisterile laboratory rearing environment, which may explain their low metabolic activity in early-instar. Not only are they related to nutritional upgrading and pathogen defense, active bacteria may also contribute to other gut metabolism, such as detoxifying plant-derived noxious allelochemicals and regulating the host's immune homeostasis. Metagenomic analysis of the labeled DNA from this study will increase our knowledge of other functions supplied by the active microbiota.

During coevolution, indigenous gut bacteria have adapted to work together in this distinct ecological niche and supply their metabolic benefits to the host. On the other hand, different physicochemical conditions with respect to host development, such as gut alkalinity, oxygen tension, might impact the community's activity, which in turn influences its composition and consequently metabolic functions. In that manner, both the host and true symbionts elaborately regulate their own metabolic activity and efficiency to preserve the host-microbial mutualism. *In vivo* activity measurement offered by SIP helps to better understand the change and maintenance of the microbial community and gives further insights into the role of active members in host fitness. Our data also established the first in-depth inventory of the gut microbiota of a model organism from mostly phytophagous Lepidoptera. Knowledge of the gut bacteria in such a major herbivore insect may also provide new targets for agricultural pest control.

This pilot study shows Pyro-SIP could rapidly gain insight into not only the structure of the community but, more important, its components' local metabolic activity with high resolution and high precision, which provides a starting point for research on more complex ecosystems, such as termite or human microbiota. Although our work has focused on the discrimination of general active gut bacteria by tracking the ubiquitous glucose, similar analysis can be performed on more specific carbon sources, for instance, labeling plant defense compounds to assess active bacteria involved in the host detoxification process. Therefore, Pyro-SIP also provides another way to understand the complex gut metabolism by breaking it down. With the development of such new approaches, a revolution in understanding of the inner world of life may come into reach soon.

## Materials and Methods

### Insect and Plant Rearing and Sample Collection

*Spodoptera littoralis* (eggs purchased from Bayer Cropscience, Monheim, Germany) were hatched and reared on a sterile artificial diet, made of white bean and essential nutrients without addition of antibiotics or preservatives. All cotton plants (*Gossypium hirsutum* DP90) were cultivated in our greenhouse under standard conditions (23±2°C; 50±5% humidity; 16 h light photo-period). To analyze the sugar composition in the gut, larvae were grown on cotton seedlings in pots or on fresh artificial diet in boxes, respectively. After 7 days of feeding, larvae were washed, sedated on ice and dissected to collect gut contents under a dissecting microscope. The alimentary canal was divided into three regions (foregut, midgut and hindgut) with sterile scissors (Fig. 1C), and gut contents from each section were released into Eppendorf tubes. Material from the same section of five to eight specimens was pooled to obtain approximately 0.6 g per sample for sugar extraction. To analyze active gut bacteria, stable isotope probing was conducted on early-instar larvae (from 1^st^ to 2^nd^) or late-instar larvae (5^th^) for 24 and 48 h using glucose-amended artificial diet. The artificial diet was frequently changed to avoid any contamination. The whole guts of larvae were collected as above for DNA extraction. For each analysis, the experiment was performed in triplicate.

### Sugar Composition Analysis

For the characterization of soluble organic compounds in the alimentary canal, freshly collected gut content was extracted according to the published literature [20], [43]. Briefly, samples were extracted with 2 ml double-distilled water in the thermomixer (Comfort, Eppendorf, Hamburg, Germany) at 60°C, 1400 rpm for approximately 2 min and subsequently cooled on ice, and homogenized with a Rotator Mixer (RM-Multi 1, STARLAB, Hamburg, Germany) for 6 h at 4°C. Supernatant fluids (extracts) were separated by centrifugation (22,000×g, 5 min at 4°C) and were filtered (0.22 µm pore size) in order to analyze the soluble sugar compounds. Because saccharides are non-volatile, a derivatization step was then conducted on the extract before GC-MS analysis as previously described [16]. After the derivativization reaction, 1 µl supernatant was subjected to GC-EIMS analysis (Finnigan Trace GC-MS 2000, ThermoQuest, Egelsbach, Germany). A Phenomenex ZB-5 column (15 m×0.25 mm, film thickness 0.25 µm) was equipped to separate sugar derivatives. Helium was used as a carrier gas at a flow rate of 1.5 ml min^−1^. The temperature program was set as follows: 80°C (2 min), then at 15°C min^−1^ to 300°C (6 min). Data were acquired and processed using the software Xcalibur (Thermo Scientific, Sunnyvale, CA, USA). Mass spectra were taken in the selected ion monitoring (SIM) mode at 70 eV. All compounds were identified by comparing their retention times and MS data with authentic references. An aliquot of the extract was also hydrolyzed with 2 M trifluoroacetic acid (TFA; Sigma-Aldrich, Saint Louis, MO, USA) at 100°C for 2 h, solubilizing the matrix polysaccharides into their monosaccharides, and subsequently derivatized to corresponding aldononitrile-acetates. Further GC-MS analysis was identical to those of non-hydrolyzed extracts. Glucose concentration was verified using a glucose assay kit based on the specific and sensitive enzymatic method according to the protocol supplied by the manufacturer (GAHK-20, Sigma-Aldrich).

### DNA Extraction and Amplification

All freshly collected gut samples were dried at 45°C in a Speedvac (Concentrator 5301, Eppendorf) and crushed in a 1.5 ml Eppendorf tube with a sterile plastic pestle. Genomic DNA was extracted using the PowerSoil™ DNA Isolation Kit (MO BIO Laboratories, Carlsbad, CA, USA) according to the manufacturer's protocol. An additional heating step at 65°C for 10 min was included just prior to bead-beating. After purification, DNA concentrations were quantified with the NanoVue spectrophotometer (GE Healthcare Europe GmbH, Freiburg, Germany). The successful extraction of microbial metagenomic DNA from the gut was verified by using PCR assays with general bacterial 16S rRNA primers (27f and 1492r) [30]. The PCR reaction was described previously [11]. Archaea- and fungus-specific primers were used to amplify archaeal 16S or fungus ITS genes (Table S1) [44]. Subsequently, the extracted DNA was used for IRMS measurement and density-gradient ultracentrifugation.

### Measurement of ^13^C Enrichment in DNA by IRMS

Carbon isotopic ratio can be a reliable indicator of the ^13^C enrichment in extracted nucleic acids. The ^13^C composition was determined by a coupled elemental analyzer/isotope ratio mass spectrometry (EA/IRMS). Purified DNA samples were placed into 0.04 ml tin capsules (3.5×5 mm, HEKAtech GmbH, Wegberg, Germany) and dried overnight. Samples were completely converted to CO_2_, N_2_ and H_2_O after combustion (oxidation at 1020°C, reduction at 650°C) in a constant helium flow (80 ml min^−1^) by using an Elemental Analyzer (EuroEA CN2 dual, HEKAtech GmbH). After passing a water trap (MgClO_4_), the gases were separated chromatographically at 85°C and transferred via the split valve to a coupled isotope ratio mass spectrometer (IRMS) (IsoPrime, Micromass, Manchester, UK). The acetanilide standard was calibrated against the international standard Vienna Pee Dee Belemnite (VPDB) using NBS 22 (IAEA reference material) with a δ^13^C value of −30.03‰ [45]. Empty tin capsules were used as blanks. Each sample was analyzed in triplicate. Isotopic ratios are expressed in delta notation as follows [46]:
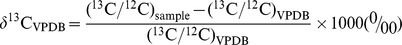



### Separation of ^13^C-labeled DNA by Density

The density-dependent resolution of the extracted DNA was conducted in cesium chloride (CsCl, Sigma-Aldrich) solution with an average density of 1.725 g ml^−1^ using a published protocol [8]. In short, approximately 1500 ng purified DNA was loaded in the CsCl centrifugation medium and filled into a 5.1 ml Quick-Seal tube (Beckmann, Fullerton, CA, USA) and sealed. All samples were set up with the same batch of CsCl medium and run in parallel to minimize potential variations. ^13^C-labeled DNA was separated by isopycnic ultracentrifugation under 50,000 r.p.m. in a NVT90 rotor in an Optima L-90K ultracentrifuge (Beckmann) at 20°C for 40 h with vacuum and subsequently the formed density gradients were fractionated from bottom to top into 12 equal fractions (425 µl each) by displacement from above with water using a HPLC pump at a flow rate of 850 µl min^−1^ (Agilent HP1100 system, Waldbronn, Germany) (Fig. 2A). Fraction density was determined by weighing an aliquot of each fraction and the last fraction containing water was excluded from the gradient fraction analysis by density. Afterwards, DNA was precipitated from every fraction, redissolved in nuclease-free water and quantified as above for the following microbial community analysis. The distribution of DNA within a given sample was calculated as the amount of the respective fraction divided by the total amount of DNA of all fractions within the sample to facilitate comparison between the gradients.

### Fingerprinting of 16S rRNA Gene in Gradient Fractions by DGGE

Separated gradient fractions were screened for differences in bacterial community composition by denaturing gradient gel electrophoresis (DGGE) analysis. Bacterial 16S rRNA gene was amplified from all fractions in two steps as a nested PCR with the primer set 27f/1492r and 968F/1401R (Table S1). DGGE was performed with the DCode system (Bio-Rad, Munich, Germany). The roughly equal amounts of PCR products (300 ng) were loaded onto the 8% polyacrylamide gel with a 20 to 80% denaturant gradient (100% denaturant was 7 M urea and 40% (v/v) deionized formamide). Electrophoresis was carried out in 1×TAE buffer at 100 V for 16 h at 60°C and the gels were stained for 30 min in 0.5×TAE buffer with SYBR-Gold nucleic acid gel stain (Invitrogen, Karlsruhe, Germany) for photographing. The DGGE patterns were compared to assess the variability of the bacterial community structure between the labeled treatment and the control. As DGGE profiles showed the difference in gradients of the labeled treatment, the respective fractions were combined to obtain one heavy (4–5), one middle (7) and one light (9–11) DNA density fraction from every sample.

### Bacterial Tag-encoded FLX Amplicon Pyrosequencing (bTEFAP) and Molecular Phylogenetic Analysis

Representative gradients were submitted to pyrosequencing as described previously [47], [48]. Basically, the hypervariable V1–V3 portion in the 16S rDNA was amplified using a Gray28F/519r primer pair and sequenced using the Roche 454 FLX Titanium based strategy (Table S1). The software package Quantitative Insight into Microbial Ecology (QIIME, 1.4.0 version) was used to process sequencing data and to calculate diversity [49], [50]. Sequences were first passed through the quality-control to remove potential artifacts and errors (the denoise_wrapper.py script was used in our analysis) and trimmed of the part with low quality [51]. Later chimera (detection method: ChimeraSlayer) and low abundance reads (<0.1%) were removed from analysis [52]. The high-quality reads were clustered into operational taxonomic units (OTUs) using UCLUST with 97% similarity cut-offs [53]. For each OTU, one representative sequence was extracted and the RDP classifier was used to determine the highest resolution of taxonomy based on the Ribosomal Database Project. Finally, an OTU table was generated describing the occurrence of bacterial phylotypes within the sample. Representative sequences were aligned to reference sequences obtained from the NCBI nucleotide database using the ClustalW algorithm. Identification of OTUs that were significantly different in abundance was carried out in METASTATS using the nonparametric *t*-test against the taxonomic data extracted from QIIME. The significance level to threshold (P value) was set at 0.05 [54]. Phylogenetic trees were calculated using the Maximum Likelihood method (Tamura-Nei model) and the Neighbor-Joining method with 1000 bootstrap replicates in MEGA5 [55].

### Fluorescence *in situ* Hybridization (FISH)

To localize the dominant gut symbionts, FISH was performed on 5 µm thin cross sections of the cold polymerizing resin (Technovit 8100, Heraeus Kulzer GmbH, Wehrheim, Germany) embedded gut tissue. The specificity of probes was tested and hybridization condition was achieved as described [11]. Shortly, the slide was hybridized with 1.5 mM of each probe (Table S1) in hybridization buffer containing 900 mM NaCl, 20 mM Tris-HCl (pH 8.0), 20% formamide, 1% SDS. FITC-labeled general eubacteria probe and Cy3-labeled *Enterococcus*-specific probe was used for detection and images were taken with an Axio Imager Z1 microscope (Carl Zeiss, Jena, Germany) [56]–[58].

### Antibiotic Bioassays

*Enterococci* were isolated from cotton leafworm gut by *Enterococcus*-selective agar plate (Fluka, Munich, Germany) and 16S rRNA gene was amplified and sequenced. The antimicrobial activity of isolated strains against different indicator bacteria was evaluated using an agar diffusion assay. The overnight culture supernatant of an *E. mundtii* strain in MRS broth (Roth, Karlsruhe, Germany) at 30°C was adjusted to pH 7.0 and filtered through 0.22 µm PVDF membrane. Holes (diameter, 6 mm) were cut out from the BHI agar plate (Roth) inoculated with indicator strains (*Micrococcus luteus* or *Leuconostoc mesenteroides*) and filled with 60 µl of *E. mundtii* culture filtrates. The agar plates were incubated at 30°C for 24 h, and antimicrobial activity was detected by the formation of clearance zones around loading holes.

### Nucleotide Sequence Accession Numbers

The pyrosequencing data have been deposited at the NCBI GenBank Short Read Archive under accession no. SRA057979.

## Supporting Information

Table S1 **Primers and probes used for the characterization and localization of bacterial taxa in the gut of** ***Spodoptera littoralis*** **larva.**
(DOCX)

Figure S1 **Sugar composition of the gut content after acid hydrolysis.** Larvae fed on cotton plants.
(TIF)

Figure S2 **Sugar composition of the gut content after acid hydrolysis.** Larvae fed on artificial diet.
(TIF)

Figure S3 **The DGGE fingerprinting of density-revealed gradient fractions from the ^13^C-glucose labeling and the native glucose control (^12^C-glucose).** (a) Fraction-dependent PCR assay of amplifying bacterial 16S rRNA gene. Fractions (1–12) were obtained from density-revealed gradients of the labeling treatment (+^13^C) or the control (+^12^C). Increased band intensity was observed in heavy fractions of the labeling treatment. (b) The DGGE profile of bacterial 16S rRNA genes in gradients of the labeling treatment and (c) the control. Arrows indicate noticeable changes in community composition of the labeling treatment.
(TIF)

Figure S4 **Fungi and archaea detection in the gut of** ***Spodoptera littoralis*** **.** Gel electrophoresis shows the absence of amplification products with fungus- and archaea-specific primers. Three kinds of common fungi-growing agar plates (KM, Kempler-McKay agar; PDA, potato dextrose agar; PNM, plant nutrient medium) were used in the fungal cultivation attempt and no fungus was recovered.
(TIF)

Figure S5 **Images of** ***Enterococcus*** **sp. from** ***S. littoralis*** **reveal bacterial gut localization and antimicrobial activity.** (a) FISH with a Cy3-labeled *Enterococcus*-specific probe (yellow) show a high density of bacterial cells adhere on the mucus layer lining the gut epithelium, and that under higher magnification (63×10) (b). White arrow indicates the gut epithelium tissue. White arrowhead indicates the gut lumen. (c) Agar diffusion assays show *Enterococcus* culture filtrates against *Micrococcus luteus* and (d) *Leuconostoc mesenteroides*. Antimicrobial activity is detected by the formation of clearance zone around the loading hole.
(TIF)

Figure S6 **The gut microflora of** ***S. littoralis*** **larvae fed on the white bean diet.** The metagenomic DNA was extracted from the whole gut tissue and sequenced without the cesium gradient ultracentrifugation.
(TIF)
