# Supplementary material for: In Vivo Pyro-SIP Assessing Active Gut Microbiota of the Cotton Leafworm, Spodoptera littoralis
Source: PLoS One. 2014 Jan 27;9(1):e85948. doi: 10.1371/journal.pone.0085948 (PMC3903505; doi:10.1371/journal.pone.0085948)
Supplement: Table S1 — Primers and probes used for the characterization and localization of bacterial taxa in the gut of Spodoptera littoralis larva. (DOCX) [file pone.0085948.s001.docx]

**Table S1.** Primers and probes used for the characterization and localization of bacterial taxa in the gut of *Spodoptera littoralis* larva.

| **Primer/Probe** |  | **Primer sequence (5'-3')** |  | **Target** |  | **Use** |  | **References** |
| --- | --- | --- | --- | --- | --- | --- | --- | --- |
|  |  |  |  |  |  |  |  |  |
| 27f |  | AGAGTTTGATCCTGGCTCAG |  | Eubacteria |  | General amplification |  | Egert *et al.* 2005 |
| 4fa |  | TCCGGTTGATCCTGCCRG |  | Archaea |  | General amplification |  |  |
| 1492r |  | GGTTACCTTGTTACGACTT |  | Eubacteria |  | General amplification |  |  |
|  |  |  |  |  |  |  |  |  |
| ITS1 |  | TCCGTAGGTGAACCTGCGG |  | Fungi |  | General amplification |  | Anderson *et al.* 2004 |
| ITS4 |  | TCCTCCGCTTATTGATATGC |  | Fungi |  | General amplification |  |  |
|  |  |  |  |  |  |  |  |  |
| M13F |  | GTAAAACGACGGCCAG |  | Eubacteria |  | Sequencing |  |  |
| M13R |  | CAGGAAACAGCTATGAC |  | Eubacteria |  | Sequencing |  |  |
|  |  |  |  |  |  |  |  |  |
| 968F-GC-Clamp | | CGCCCGGGGCGCGCCCCGGGCGGGGCGGGGGCACGGGGGGAACGCGAAGAACCTTAC | | Eubacteria |  | DGGE |  | van Ems *et al.* 2008 |
| 1401Ra |  | CGGTGTGTACAAGGCCCGGGAACG |  | Eubacteria |  | DGGE/Sequencing |  |  |
| 1401Rb |  | CGGTGTGTACAAGACCCGGGAACG |  | Eubacteria |  | DGGE/Sequencing |  |  |
| 968F |  | AACGCGAAGAACCTTAC |  | Eubacteria |  | Sequencing |  |  |
|  |  |  |  |  |  |  |  |  |
| Gray28F |  | GAGTTTGATCNTGGCTCAG |  | Eubacteria |  | 454 sequencing |  | Ishak *et al.* 2011 |
| Gray519r |  | GTNTTACNGCGGCKGCTG |  | Eubacteria |  | 454 sequencing |  |  |
|  |  |  |  |  |  |  |  |  |
| EUB338-Cy5 |  | GCTGCCTCCCGTAGGAGT |  | Eubacteria |  | FISH |  | Amann *et al.* 1990 |
| CGF -Cys3 |  | GCGGAAAATAGTGTTATACGG |  | *Enterococccus spp.* |  | FISH |  | Manero *et al.* 2002 |
| Ecf459-Cy3 |  | GGGATGAACATTTTACTC |  | *Enterococccus spp.* |  | FISH |  | Behr *et al.* 2000 |
|  |  |  |  |  |  |  |  |  |
|  |  |  |  |  |  |  |  |  |
