# Supplementary figures and images for: In Vivo Pyro-SIP Assessing Active Gut Microbiota of the Cotton Leafworm, Spodoptera littoralis
Source: PLoS One. 2014 Jan 27;9(1):e85948. doi: 10.1371/journal.pone.0085948 (PMC3903505; doi:10.1371/journal.pone.0085948)

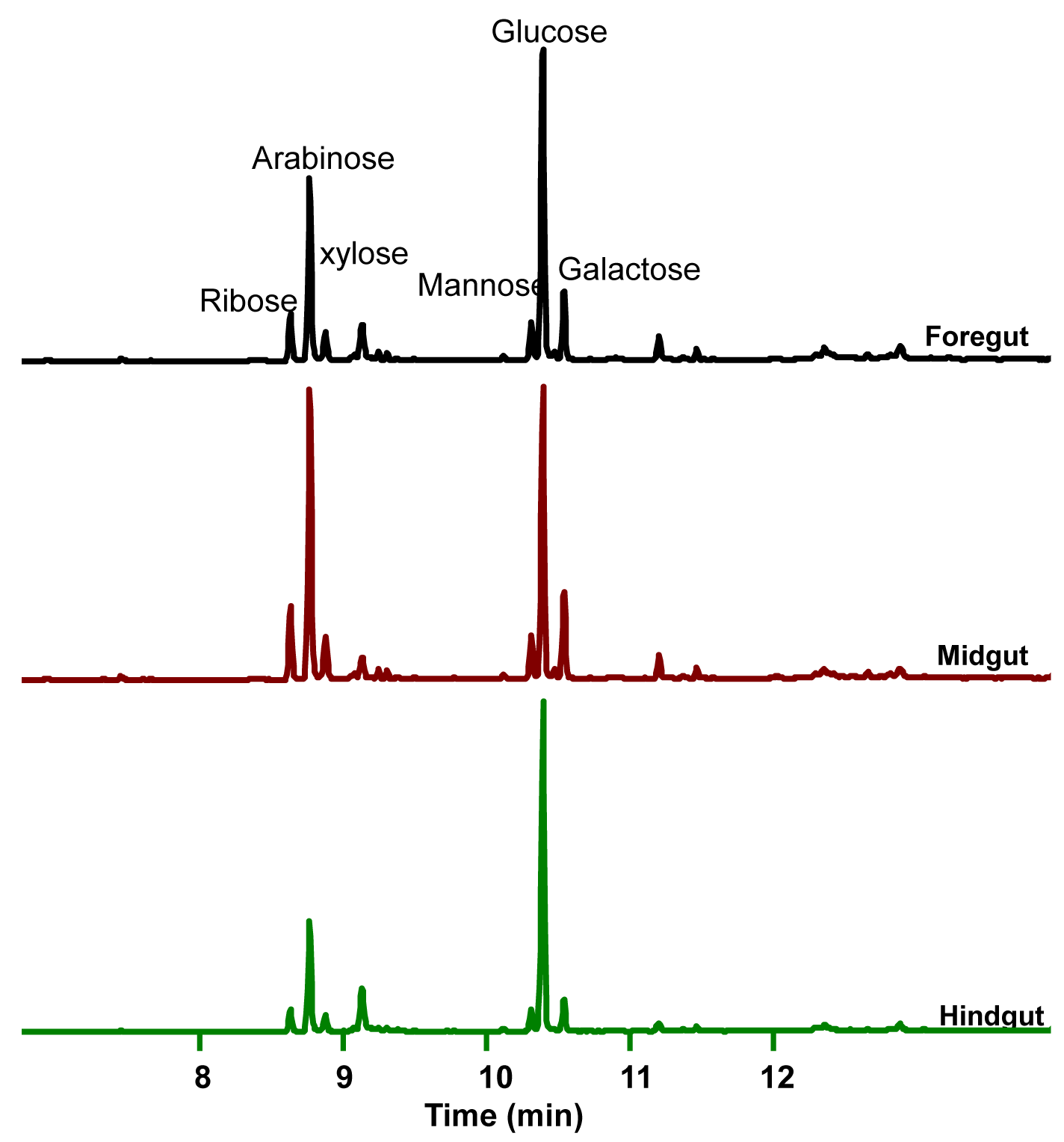

Supplement: Figure S1 — Sugar composition of the gut content after acid hydrolysis. Larvae fed on cotton plants. (TIF) [file pone.0085948.s002.tif]

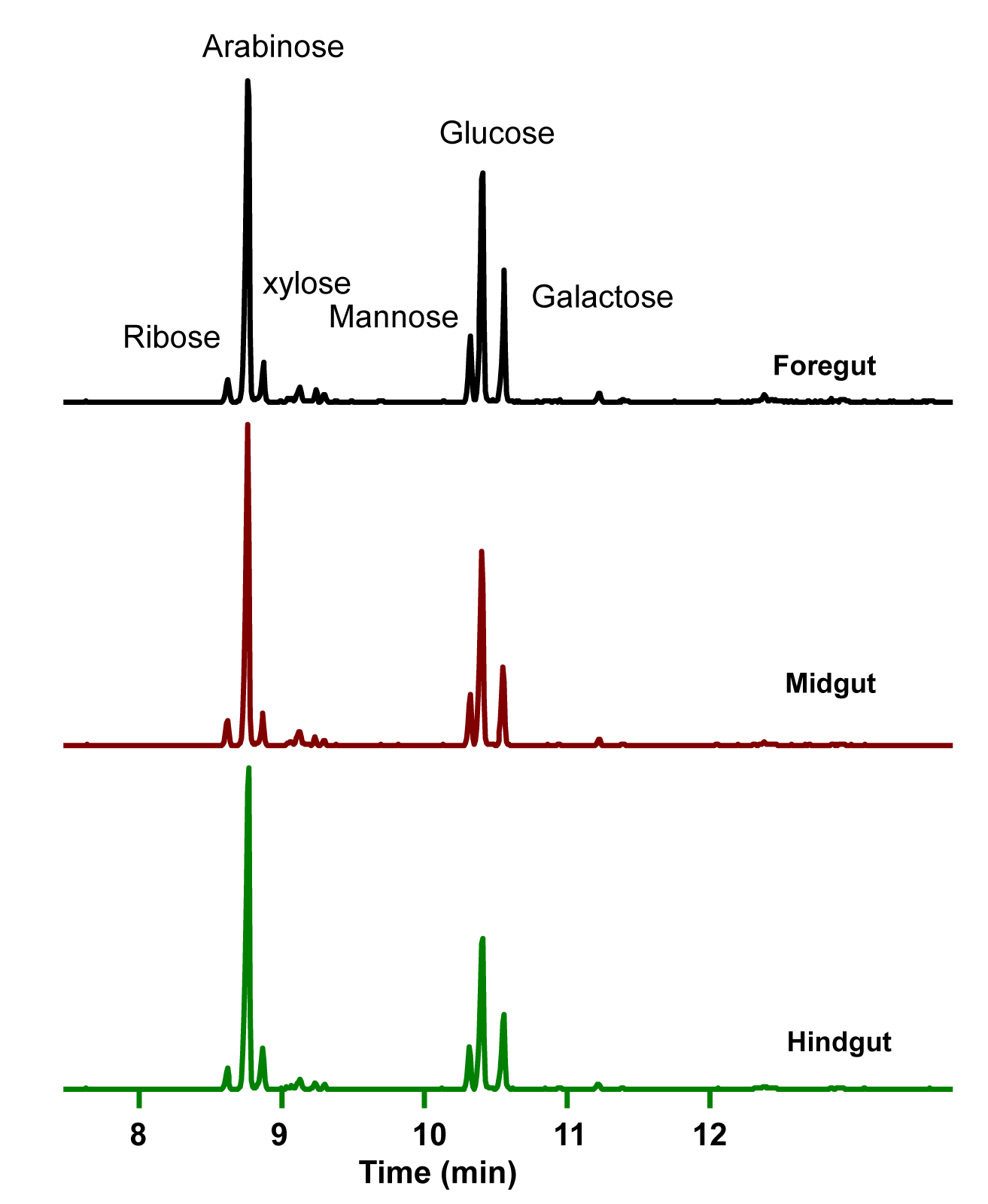

Supplement: Figure S2 — Sugar composition of the gut content after acid hydrolysis. Larvae fed on artificial diet. (TIF) [file pone.0085948.s003.tif]

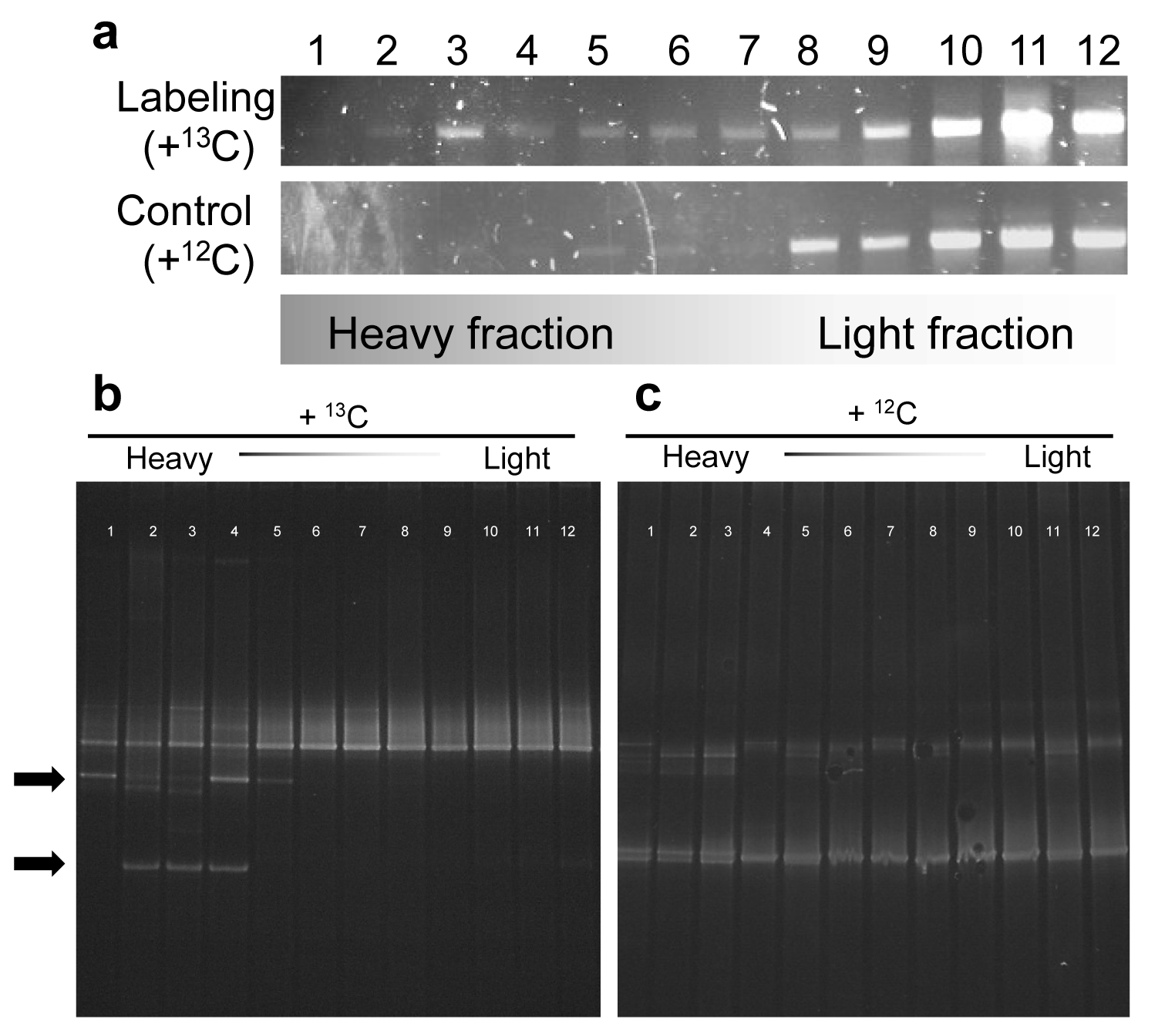

Supplement: Figure S3 — The DGGE fingerprinting of density-revealed gradient fractions from the 13C-glucose labeling and the native glucose control (12C-glucose). (a) Fraction-dependent PCR assay of amplifying bacterial 16S rRNA gene. Fractions (1–12) were obtained from density-revealed gradients of the labeling treatment (+13C) or the control (+12C). Increased band intensity was observed in heavy fractions of the labeling treatment. (b) The DGGE profile of bacterial 16S rRNA genes in gradients of the labeling treatment and (c) the control. Arrows indicate noticeable changes in community composition of the labeling treatment. (TIF) [file pone.0085948.s004.tif]

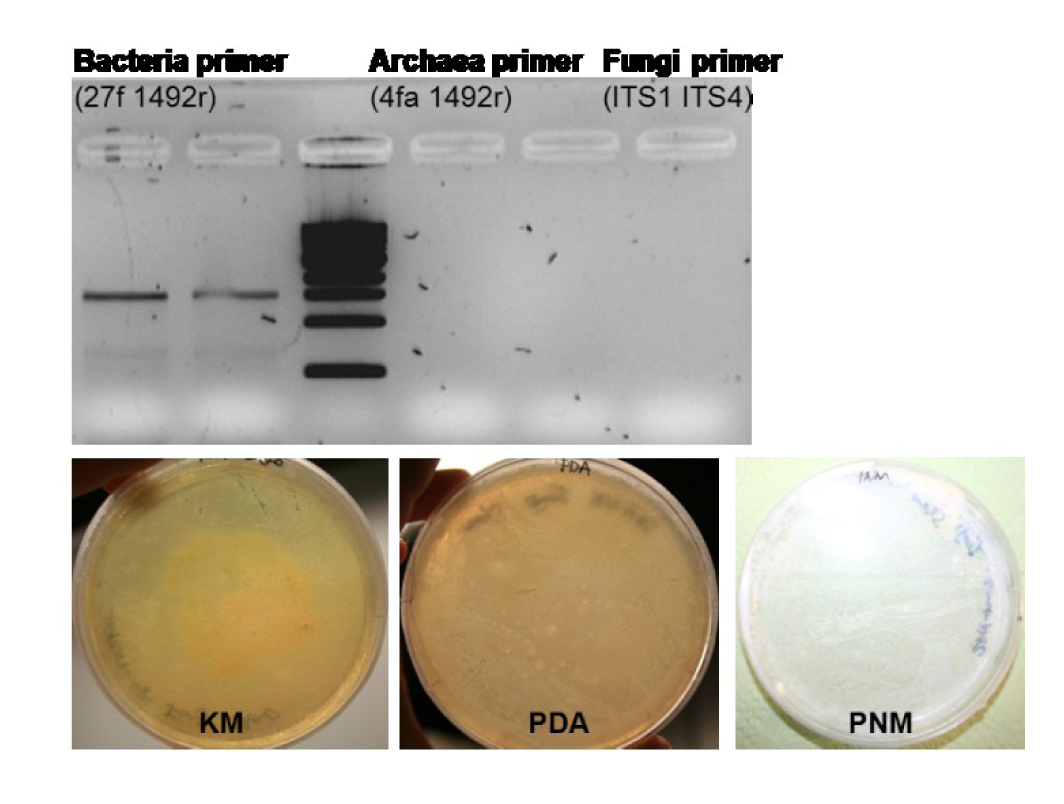

Supplement: Figure S4 — Fungi and archaea detection in the gut of Spodoptera littoralis . Gel electrophoresis shows the absence of amplification products with fungus- and archaea-specific primers. Three kinds of common fungi-growing agar plates (KM, Kempler-McKay agar; PDA, potato dextrose agar; PNM, plant nutrient medium) were used in the fungal cultivation attempt and no fungus was recovered. (TIF) [file pone.0085948.s005.tif]

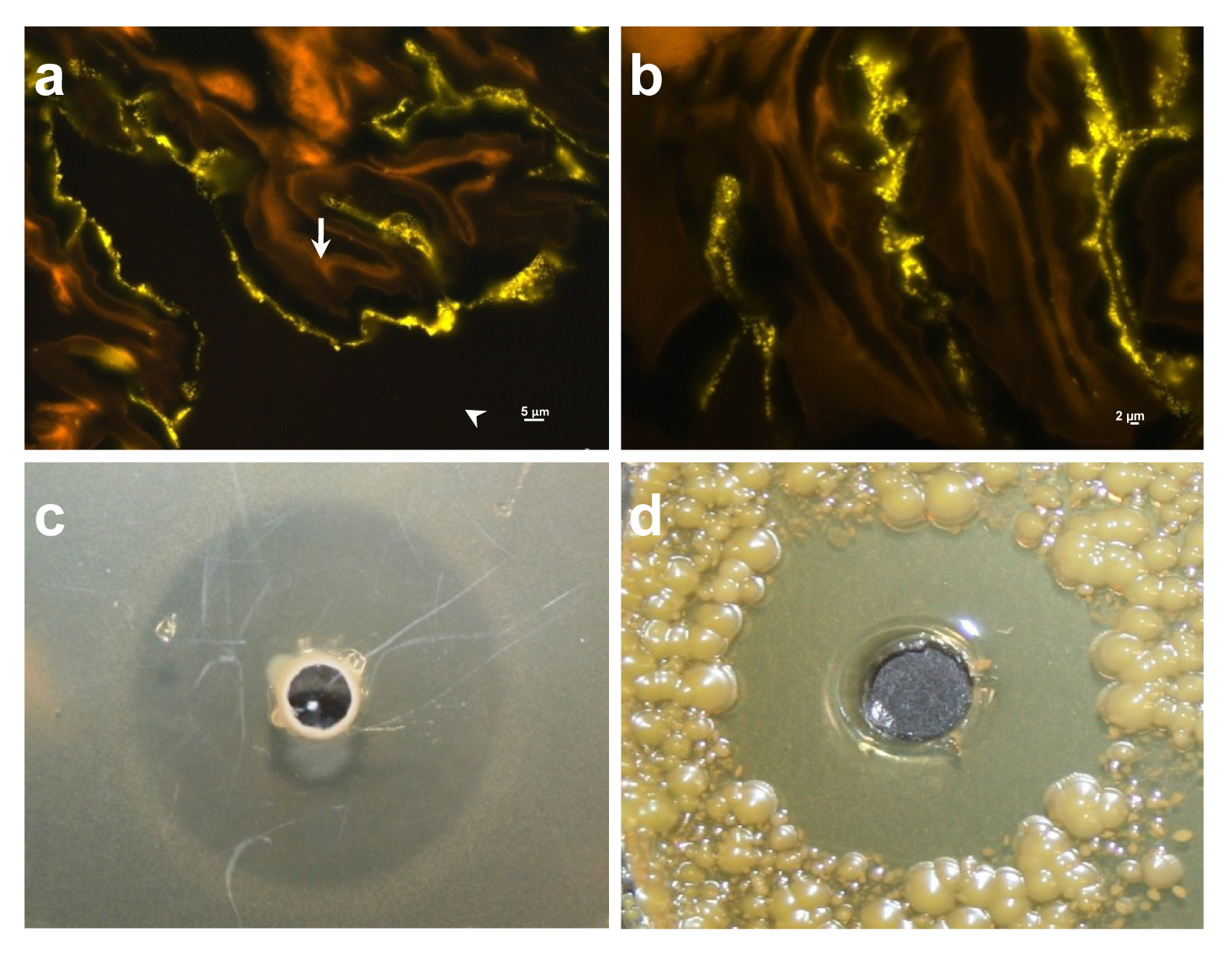

Supplement: Figure S5 — Images of Enterococcus sp. from S. littoralis reveal bacterial gut localization and antimicrobial activity. (a) FISH with a Cy3-labeled Enterococcus-specific probe (yellow) show a high density of bacterial cells adhere on the mucus layer lining the gut epithelium, and that under higher magnification (63×10) (b). White arrow indicates the gut epithelium tissue. White arrowhead indicates the gut lumen. (c) Agar diffusion assays show Enterococcus culture filtrates against Micrococcus luteus and (d) Leuconostoc mesenteroides. Antimicrobial activity is detected by the formation of clearance zone around the loading hole. (TIF) [file pone.0085948.s006.tif]

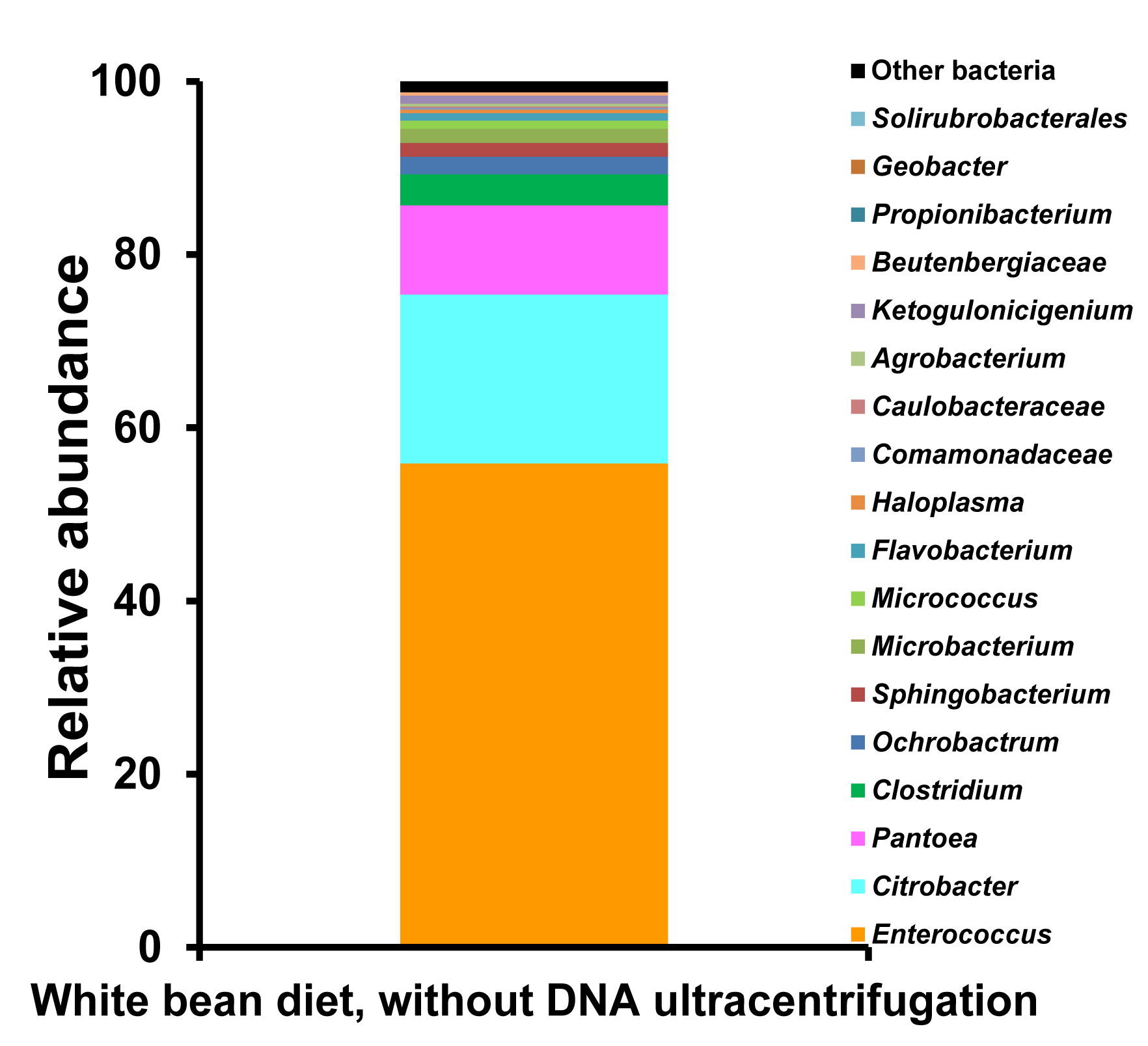

Supplement: Figure S6 — The gut microflora of S. littoralis larvae fed on the white bean diet. The metagenomic DNA was extracted from the whole gut tissue and sequenced without the cesium gradient ultracentrifugation. (TIF) [file pone.0085948.s007.tif]
